# Editorial: Antioxidant and neuroprotective potential of alternative and complementary therapeutic approaches against Alzheimer’s disease

**DOI:** 10.3389/fphar.2023.1289979

**Published:** 2023-10-19

**Authors:** Sandeep Kumar Singh, Alessandra Durazzo, Massimo Lucarini, Soraya L. Valles, Burkhard Poeggeler

**Affiliations:** ^1^ Indian Scientific Education and Technology Foundation, Lucknow, India; ^2^ CREA-Research Centre for Food and Nutrition, Rome, Italy; ^3^ Department of Physiology, School of Medicine, University of Valencia, Valencia, Spain; ^4^ Faculty of Biology and Psychology, Johann-Friedrich-Blumenbach-Institute for Zoology and Anthropology, Georg August University Göttingen, Göttingen, Germany

**Keywords:** alternative and complementary medicine, Alzheimer’s disease, antioxidant, ayurvedic, herbal compounds, inflammaging, neuroprotection, oxidative stress

In the collection of articles under the Research Topic: “*Antioxidant and neuroprotective potential of alternative and complementary therapeutic approaches against Alzheimer’s disease,*” five articles have been published.

The data and findings presented in this article Research Topic on the *Antioxidant and neuroprotective potential of alternative and complementary therapeutic approaches against Alzheimer’s disease* show how molecular mechanisms and mediators can be targeted to provide antioxidant and neuroprotective effects, protecting against Alzheimer’s disease.

Alzheimer’s disease is a progressive neurodegenerative disease that is characterized by the impairment of memory and cognitive function. Icariin is a natural compound isolated from Epimedii herba that can protect against Alzheimer’s disease (You et al.). A comprehensive review of preclinical studies coupled with network pharmacology demonstrates that icariin could protect against the neurodegeneration associated with Alzheimer’s disease by regulating the expression of Aβ_1-42_, Aβ_1-40_, BACE1, tau, hyperphosphorylated tau, and inflammatory mediators. The work identifies 35 specific molecular targets with the HIF-1 signaling pathway ranking first according to the Kyoto Encyclopedia of Genes and Genomes pathway analysis. Icariin effectively docked to the 35 hub targets including HIF-1α, and the binding of the HIF-1-Icariin complex within 100 ns which indicates that icariin contributes to the stability of HIF-1α (You et al.). The HIF-1 signaling pathway seems to be an important target that can mediate the neuroprotective effects of icariin in Alzheimer’s disease.

The Huanglian Jiedu decoction is a Chinese herbal formula that exerts neuroprotective effects by protecting against oxidative stress in Alzheimer’s disease and its neuroprotective effects were screened through a novel magnetic nanoparticle-assisted cell membrane chromatography method by using immobilized stable amide bonds (Liao et al.). Fifteen components were found to specifically bind to cell membranes, and seven of them reduced glutamate-induced toxicity in HT-22 cells (Liao et al.). Amide bond-based immobilization of magnetic nanoparticles on cell membranes, along with solid-phase extraction and ultraperformance liquid chromatography, can be employed successfully for the isolation and discovery of the bioactive components in such decoctions.

Another study (Su et al.) is focused on Radix Rehmanniae Praeparata being used as a medicine in Chinese herbal formula for the treatment of Alzheimer’s disease. Radix Rehmanniae Praeparata protected against cognitive dysfunction and pathological changes of brain tissue of mice treated intracerebroventricular with streptozotocin, a well-established Alzheimers’s disease animal model, as demonstrated by reduced tau protein hyperphosphorylation, expression levels of insulin receptors, IRS-1, pSer473-AKT/AKT, and pSer9-GSK-3β/GSK-3β levels in hippocampal and cortical tissues (Su et al.). Radix Rehmanniae Praeparata also prevented the dysregulation of intestinal microbiota induced by streptozotocin in mice. Mass spectrometry analysis showed that the Radix Rehmanniae Praeparata contained seven compounds, Acteoside (Verbascoside), 5-Hydroxymethyl-2-furaldehyde (5-HMF), Apigenin-7-O-glucuronide, Icariin, Gallic acid, Quercetin-3β-D-glucoside, and Geniposide. These molecules may mediate the potent neuroprotection.

The next study (Foudah et al.) aimed to investigate how resveratrol (RES) alone and in combination with vitamin E affected rats with Alzheimer’s disease using scopolamine. Scopolamin-induced changes in rats were reduced after resveratrol treatment (Foudah et al.). Acetylcholinesterase, protein carbonyl, and TNF-α improved after resveratrol treatment. RES increased the levels of antioxidants, decreased Scopolamin-induced lipid peroxidation, and reversed Scopolamin-mediated biochemical and behavioral changes comparable to that of drugs such as donepezil. Vitamin E showed a synergistic effect on resveratrol in limiting cognitive impairment (Foudah et al.).

Furthermore, the study by Lu et al. reports how *Jasminum grandiflorum* L. essential oil not only significantly reverses the proinflammatory changes such as microglia activation but also inhibits the formation of nitric oxide and reactive oxygen species and suppresses tumor necrosis factor-α, interleukin, and ionized calcium-binding adapter molecule 1 expression (Lu et al.).

The research conclusively demonstrates that antioxidant and antiinflammatory agents can exert potent neuroprotective effects against Alzheimer’s disease. Decisive molecular mechanisms and mediators have been identified that can be targeted by innovative approaches to the prevention and treatment of this neurodegenerative disease. Complementary and alternative medicine has the potential to protect the brain against damage and to prevent the associated cognitive decline.

